# *Spermacoce alata* Aubl. Essential Oil: Chemical Composition, In Vitro Antioxidant Activity, and Inhibitory Effects of Acetylcholinesterase, α-Glucosidase and β-Lactamase

**DOI:** 10.3390/molecules29122869

**Published:** 2024-06-16

**Authors:** Xinyu Zhu, Jiadong Zhu, Ziyue Xu, Xu Liu

**Affiliations:** 1 SDU-ANU Joint Science College, Shandong University, Weihai 264209, China; 202200700229@mail.sdu.edu.cn (X.Z.); 202000700248@mail.sdu.edu.cn (J.Z.); zxudk@connect.ust.hk (Z.X.); 2 Department of Ocean Science, The Hong Kong University of Science and Technology, Clear Water Bay, Kowloon, Hong Kong SAR 999077, China; 3 Marine College, Shandong University, Weihai 264209, China

**Keywords:** *Spermacoce alata* Aubl., essential oil, chemical composition, antioxidant, anti-acetylcholinesterase, anti-α-glucosidase, anti-β-lactamase

## Abstract

*Spermacoce alata* Aubl. is widely available in the market as traditional Chinese medicine and animal feed, due to its properties of clearing heat and treating malaria and its high-protein and crude fiber content. In this study, the essential oil of *S. alata* was obtained through hydrodistillation. GC–MS and GC–FID methods were used to identify the chemical components and their relative abundance. Furthermore, the antioxidant capacity was measured using DPPH, ABTS, and FRAP assays, and the inhibitory effects of acetylcholinesterase, α-glucosidase, and β-lactamase were also evaluated. A total of 67 compounds were identified, with the major constituents being palmitic acid (30.74%), linoleic acid (16.13%), and phenylheptatriyne (8.07%). The essential oil exhibited moderate antioxidant activity against DPPH (IC_50_ > 10 mg/mL), while the IC_50_ value for the ABTS assay was 3.84 ± 2.12 mg/mL and the FRAP assay value was 87.22 ± 12.22 µM/g. Additionally, the essential oil showed moderate anti-acetylcholinesterase activity (IC_50_ = 286.0 ± 79.04 μg/mL), significant anti-α-glucosidase activity (IC_50_ = 174.7 ± 13.12 μg/mL), and potent anti-β-lactamase activity (IC_50_ = 37.56 ± 3.48 μg/mL). The results suggest that *S. alata* has the potential for application in pharmacology, warranting further exploration and investigation.

## 1. Introduction

Antioxidants are compounds that can prevent, delay, or reverse oxidation reactions by donating electrons to free radicals, thereby preventing cell dysfunction caused by free radicals [1]. They are commonly used to prevent the oxidation of lipids and proteins and have been shown to play an essential role in preventing and controlling many diseases in the body, such as diabetes mellitus, neurodegenerative diseases, inflammation, and cancer, by mitigating the adverse effects of oxidative stress [2,3]. While widely used synthetic antioxidants are effective, concerns exist regarding their potential adverse effects on human health, prompting a long-standing search for natural antioxidant sources [4]. Plant essential oils have shown promising results in this regard and are increasingly in demand as natural alternatives to synthetic antioxidants [5,6], highlighting the significance of extracting antioxidant constituents from plants.

Alzheimer’s disease, a neurodegenerative disease associated with impaired memory and other cognitive functions, is considered one of the most severe threats to older people [7]. One of the principal therapeutic approaches for Alzheimer’s disease centers on inhibiting acetylcholinesterase activity [8]. Acetylcholinesterase is primarily responsible for breaking down acetylcholine, rendering it inactive and halting nerve signaling. Therefore, acetylcholinesterase inhibitors are commonly used in the treatment of Alzheimer’s disease and other related conditions [9]. Hung et al. found that plant essential oils possess inhibitory activity against acetylcholinesterase, which could profoundly influence the treatment strategies for Alzheimer’s disease [10]. In addition, studies have also shown that oxidative stress and free radical damage may be the initial indicators of Alzheimer’s disease, and antioxidants can mitigate oxidative stress by protecting cells from Aβ-induced neurotoxicity or inhibiting the formation and stabilization of amyloid-β fragments (fAβ). Therefore, antioxidants play an essential role in managing and controlling Alzheimer’s disease [11]. Alzheimer’s disease can be treated by anti-acetylcholinesterase and antioxidant methods [12]. 

Diabetes mellitus (DM) is a common chronic metabolic disorder characterized by hyperglycemia caused by the disturbance of carbohydrate metabolism [13]. α-Glucosidase is a key glucoside hydrolase that catalyzes the hydrolysis of disaccharides and oligosaccharides into absorbable monosaccharides in the final step of carbohydrate digestion [14,15]. Therefore, glucosidase inhibitors can effectively treat diabetes mellitus by delaying the breakdown of carbohydrates, inhibiting glucose absorption, and reducing blood sugar levels. However, acarbose, the most widely used drug, can cause abdominal discomfort, such as bloating and diarrhea, in nearly 20% of patients, making it essential to find alternatives [16]. Previous studies have found that terpenoid compounds such as β-pinene, γ-terpinene, α-terpineol, and linalool from plant extracts and essential oils exhibited α-glucosidase inhibitory effects, making them potential treatments for diabetes mellitus [17,18].

β-lactam antibiotics are widely used to treat bacterial infectious diseases [19,20]. They work by forming acylase complexes with penicillin-binding proteins (PBPs), destroying the integrity of the cell wall and eventually leading to cell lysis, thereby inhibiting transpeptidase activity [21]. However, β-lactam antibiotics exert a strong selective pressure on bacteria, driving their evolution to produce a suite of enzymes capable of effectively degrading or inactivating β-lactam antibiotics, consequently endowing bacteria with increasing tolerance [22,23]. Therefore, β-lactamase inhibitors need to be urgently developed [19,24]. Hayanni and Shora found that various natural products from plants have an inhibitory effect on β-lactamase activity [25]. Therefore, the extraction of β-lactamase inhibitors from plants might offer a potential treatment strategy for bacterial infections resistant to β-lactam antibiotics.

Plant essential oils, derived from flowers, fruits, stems, roots, and other parts of plants, are natural substances with a wide range of biological activities, such as anti-acetylcholinesterase activity, anti-α-glucosidase activity, antibacterial activity, and antioxidant properties, which have promoted their application in the pharmacology, cosmetics, and food industries [26,27]. Essential oils have been used for traditional medicinal purposes since antiquity, and the recent rise in interest in green consumerism has led to a preference for natural products over synthetic ones [28]. In addition to their medicinal value, plant essential oils can be applied in the breeding industry to maintain animal health, enhance animal production capacity, and improve the quality of livestock products [29]. Given the practical value of essential oils, further exploration of their potential capacity extracted from natural plants is warranted.

The genus *Spermacoce* comprises approximately 250 to 300 species distributed in tropical and subtropical regions [30]. It is found in Mexico, South America, Africa, Asia, and Australia [31]. *Spermacoce alata* is an herbaceous plant with pubescent stems, elliptical leaves, smooth surfaces, and membranous stipules. Known for clearing heat, detoxication, and high-quality feeding value, *S. alata* has broad application prospects. As a traditional medicine, *S. alata* treats malaria in Nepal [32]. In Nigeria, they are used to treat human schistosomiasis [33]. The compounds of *S. alata* can reduce chronic low-grade inflammation, hepatic lipid toxicity, oxidative stress, and insulin resistance by regulating the activity of metabolic transcription factors and taking advantage of the prebiotic activity, free radical-scavenging ability, and immunomodulatory properties of secondary metabolites to prevent and treat metabolic syndromes such as diabetes mellitus, fatty liver disease, atherosclerosis, and cardiovascular disorders, which have broad therapeutic prospects [34,35]. In previous studies, it has also exhibited potent anti-leukemia activity [36]. In agricultural production, *S. alata* can also be used as feed for livestock, with studies showing that adding *S. alata* to feed can significantly enhance the antioxidant capacity of chicken, extend the shelf life of meat products, and positively influence overall broiler production [37].

The clinical effectiveness and practical applications of *S. alata* primarily result from the various biological activities of its chemical components. However, no comprehensive investigation was conducted on the essential oil of *S. alata*. Therefore, the present study aims to investigate the antioxidant activity, anti-acetylcholinesterase, anti-α-glucosidase, and anti-β-lactamase activities of essential oil from *S. alata*.

## 2. Results and Discussion

### 2.1. Essential Oil Yield and Component Analysis

**Figure 1:**
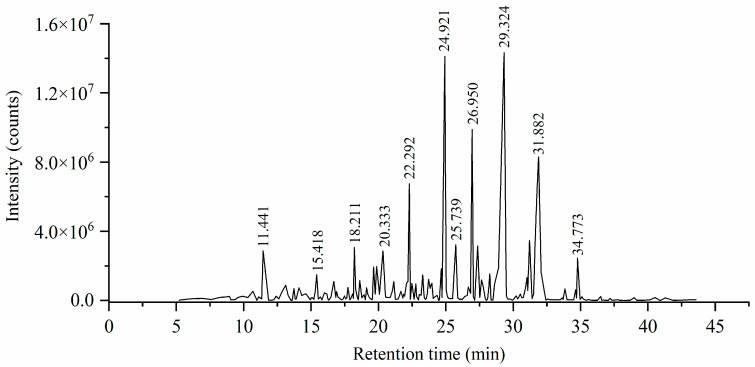
The total ion chromatogram of *S. alata* essential oil derived from GC–MS.

The essential oil of *S. alata* obtained by hydrodistillation was a green hydrophobic oily liquid. The average yield of essential oil was 0.10 mL/kg. In previous studies of the Rubiaceae family plants, the yields of the essential oils of *R. tinctorum*, *C. glabra*, and *P. leiocarpa* were 0.10 mL/kg, 0.08 mL/kg, 0.10 mL/kg, respectively [38,39,40]. Moreover, the latest study found that five species of Rubiaceae plants yielded 0.24 mL/kg, 0.32 mL/kg, 0.08 mL/kg, 0.05 mL/kg, and 0.30 mL/kg, respectively [41]. Collectively, the yield of our essential oil is similar to those mentioned above, which is consistent with the Rubiaceae-characteristic yield. The total ion chromatogram (TIC) of *S. alata* is shown in Figure 1.

The retention time (RT), retention index (RI), and percentage (%) of this essential oil are listed in Table 1 according to the elution sequence on the HP-5MS column. A total of 67 compounds were identified, accounting for 95.44% of the essential oil from GC–FID (Supplementary Materials) and GC–MS analysis [42]. The main components were palmitic acid (30.74%), linolenic acid (16.13%), phenylheptatriyne (8.07%), hexahydrofarnesyl acetone (4.44%), tetradecanoic acid (3.16%), linalool (3.08%), and caryophyllene oxidate (2.69%). Among them, fatty acids account for 55.03%, sesquiterpenoids for 9.70%, and monoterpenoids for 9.49%. These results indicate that *S. alata* could be classified as a fatty acid chemotype. As previously reported, essential oils from other *Spermacoce* plants, such as *S. pusilla*, contained main compounds including palmitate (25.09%), oleic acid (7.78%), humulene (6.19%), and humulene oxide II (6.08%) [41], which also exhibited a notable abundance of fatty acids and esters. However, the main components of *S. verticillata* essential oil were phytol (56.30%), 1,8-cineole (20.40%), α-pinene (7.10%), and p-cymene (4.00%). This terpenoid chemotype is different from other species within the genus *Spermacoce*, suggesting the chemical diversity within the genus [43]. One previous study focused on the chloroform extracts of *S. alata* and identified 35 compounds, including mono (2-ethylhexyl) phthalate (21.64%), isobutyl acetate (14.62%), hexahydrofarnesyl acetone (12.66%), *n*-hexanal (12.28%), and 2-nitrocthanol (8.09%) [44]. These compounds are similar to those of the essential oil of *S. alata* analyzed in the present study, which is rich in fatty acid and esters compounds. The differences in their compound composition may be attributed to their distinct extraction methods.

In previous studies, fatty acid chemotype essential oil was considered to possess antioxidant, anti-acetylcholinesterase, and other biological activities, providing a research direction for further exploration of phytomedicine [45]. The predominate component in essential oil, palmitic acid (30.74%), a saturated fatty acid, has been reported to exhibit antibacterial activity by impairing the bacterial cell membrane, causing the leakage of cellular contents and ultimately resulting in bacterial death [46]. Additionally, low concentrations of palmitic acid exert a protective antioxidant effect in cardiomyoblasts, suggesting its potential to protect the heart from oxidative stress [47]. The second most abundant compound is linolenic acid (16.13%), an unsaturated fatty acid commonly found in nature, which can attenuate Alzheimer’s disease pathology, such as tau phosphorylation, blood–brain barrier disruption, synaptic dysfunction, and cognitive impairment. Furthermore, it can be a therapeutic agent for diabetes mellitus by enhancing mitochondrial biogenesis and modulating insulin signaling [48]. The third major component, phenylheptatriyne (8.07%) exhibits significant antibacterial activity against various strains of bacteria, laying a foundation for replacing synthetic fungicides with phenylheptatriyne [49]. The compounds in the *S. alata* mentioned above can treat various diseases, livestock breeding, and the food industry. Therefore, regarding the rich chemical composition, we further conduct experiments on the antioxidant activity, anti-acetylcholinesterase, anti-α-glucosidase, and anti-β-lactamase activities of the essential oil to explore its potential medical value.

### 2.2. Antioxidant Activity Evaluation

Free radical diphenylpicrylhydrazyl (DPPH), 2,20-azinobis (3-ethylbenzothiazoline-6-sulfonate) (ABTS), and ferric reducing activity power assay (FRAP) are the most prevalent analytical assays used in antioxidant evaluation [50]. In this study, the antioxidant activity of *S. alata* essential oil was measured by DPPH, ABTS, and FRAP assays. The antioxidant values of the three assays are presented in Table 2. 

DPPH determination is a simple, effective, and rapid method widely used in studying natural compounds to evaluate antioxidant activity [51]. The DPPH radical accepts electrons or hydrogen radicals from the donor compound and exhibits a strong absorption band at 515–520 nm [52]. At the highest concentration (10 mg/mL), the antioxidant activity of essential oil was 31.98%, whose potency seems weaker than those obtained from essential oils of other species [45,53,54].

**Figure 2:**
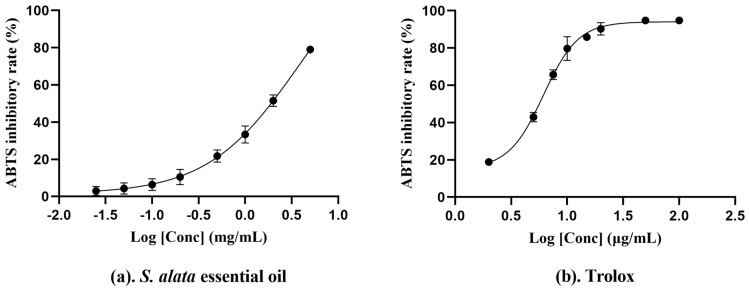
Variation in ABTS radical−scavenging percentage with varying concentrations for *S. alata* essential oil (**a**) and Trolox (**b**). The data were calculated from three parallel experiments.

The results in Figure 2 showed that the activities of essential oil and Trolox increased in a sigmoidal dose-dependent manner within the concentration range in the ABTS assay. The IC_50_ values of ABTS-scavenging capacity of our essential oil and Trolox were 3.84 ± 2.12 mg/mL and 6.1 ± 1.4 µg/mL, respectively. Prior research has shown the IC_50_ values of *A. annua* and *O. vulgare* to be at 5.97 ± 0.51 mg/mL and 7.35 ± 0.30 mg/mL, respectively [55,56], suggesting their ABTS^•+^ free radical-scavenging activities are inferior to our essential oil. Nonetheless, other essential oils, such as *S. rhombifolia* and *T. triquetrum*, have demonstrated superior ABTS^•+^-scavenging activities with IC_50_ values of 1.47 ± 0.01 mg/mL [53] and 2.12 ± 0.05 mg/mL [54], indicating a more potent ability to neutralize free radicals. In the preceding experiments, the ABTS assay consistently demonstrated superior sensitivity in detecting free radical-scavenging activity compared to the DPPH assay. The result suggests that the ABTS assay may offer enhanced precision in antioxidant activity [57].

The difference between the DPPH and ABTS assay may be attributable to slightly different mechanisms of action: DPPH is mainly based on the hydrogen atom transfer (HAT) mechanism. In contrast, the ABTS assay is primarily based on the electron transfer (ET) mechanism [58]. Furthermore, DPPH radicals are more sensitive to the reaction environment (solvent, pH, and temperature) than ABTS^•+^ radical cations, resulting in higher variability, which may be why the ABTS assay manifested a stronger ability to scavenge free radicals [59].

The FRAP assay is used to evaluate the total antioxidant capacity of antioxidants to reduce Fe (III)-TPTZ to Fe (II)-TPTZ in the presence of a low pH [60]. The absorbance increases with the formation of the Fe (II)-TPTZ complex [61]. As shown in Table 2, the essential oil of *S. alata* exhibited an antioxidant capacity of 87.22 ± 12.22 μM/g measured by the FRAP assay, surpassing other plants such as *O. basilicum* (47.88 ± 1.08 µM/g) [62], *M. coromandelianum* (63.24 ± 4.81 µM/g) [63]. Since the FRAP assay is based on the single electron transfer (SET) mechanism, it offers a distinct advantage because it is not limited to specific structural groups or compounds. Instead, it evaluates all oxidizable entities that can engage with the assay reagent. Consequently, it provides a quantifiable measure of reducing capacity, distinguishing it from methods focusing on radical-scavenging activity [59].

The antioxidant capacity is generally a multifaceted attribute. It is more worthwhile to select different methods that are not closely related to each other to understand the antioxidant mechanisms in the specific antioxidant [59].

Prior research has demonstrated that molecules capable of scavenging DPPH free radicals feature double bonds, especially those with conjugated double bonds which afford rapid and efficient scavenging activities, such as β-cyclotrienal (0.17%), β-ionic ketone (1.29%), and α-terpinol (0.39%) in this essential oil. Their conjugated double bonds can form a resonance structure with the DPPH free radical, reducing it to hydrazine and forming an antioxidant free radical, thereby terminating the free radical chain reaction [64]. Nonetheless, the relatively low abundance of such compounds may account for its diminished DPPH free radical-scavenging activity within our essential oil. In addition, phenolic compounds, such as 4-ethyl-2-methoxyanisole (0.26%) in this essential oil, can provide hydrogen in the hydroxyl group to scavenge superoxide anions, hydroxyl radicals, and other free radicals under in vitro conditions effectively [65,66]. Moreover, terpenoids containing enols have unsaturated hydroxyl groups, such as phytol (1.25%) and linalool (3.08%) in this essential oil may also exhibit intense antioxidant activity [67]. This could potentially be the principal contributor to the antioxidant ability of this essential oil.

### 2.3. Anti-Acetylcholinesterase Activity

**Figure 3:**
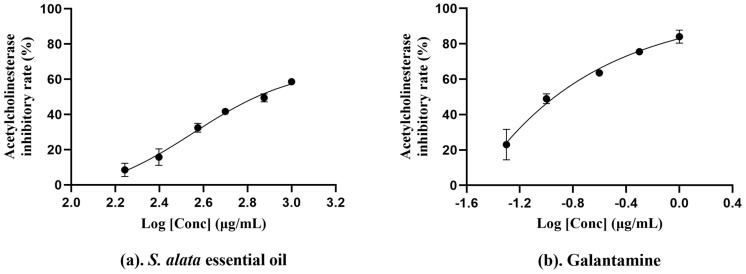
The concentration−dependent anti−acetylcholinesterase activity of *S. alata* essential oil (**a**) and Galantamine (**b**). The data were calculated from three parallel experiments.

Acetylcholinesterase inactivates neurotransmitters in cholinergic synapses by hydrolyzing acetylcholine. Therefore, compounds that possess anti-acetylcholinesterase ability are considered promising in the treatment of neurodegenerative diseases such as Alzheimer’s disease [68]. In this study, we assessed the anti-acetylcholinesterase activity of the essential oil. As detailed in Table 3, the essential oil demonstrated an IC_50_ value of 286.0 ± 79.04 µg/mL, indicating a stronger anti-acetylcholinesterase activity than essential oil from *C. limon* (849.90 ± 11.50 μg/mL) and *F. vulgare* (1187.7 ± 11.50 µg/mL) [69] but weaker than essential oil from *L. nervosa* (51.96 ± 14.26 µg/mL) and *O. majorana* (150.33 ± 2.02 µg/mL) [45,66], suggesting that this essential oil possesses moderate anti-acetylcholinesterase activity. Previous studies have shown that the anti-acetylcholinesterase activity of essential oil can be attributed to monoterpenoids or oxygen-containing monoterpenoids, especially the bicyclic monoterpenoids containing allyl methyl groups [66]. In another study, linalool is one of the monoterpenoids with the most vigorous acetylcholinesterase inhibitory activity [70]. Therefore, we speculated that linalool (3.08%), camphol (0.56%), and nerol (0.33%) may be the sources of anti-acetylcholinesterase activity (Figure 3).

### 2.4. Anti-α-Glucosidase Activity

**Figure 4:**
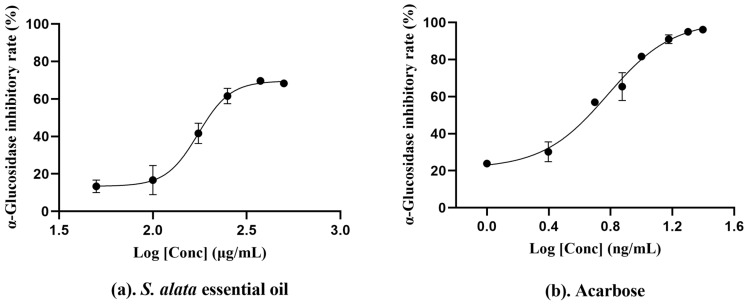
The concentration−dependent anti−α−glucosidase activity of *S. alata* essential oil (**a**) and Acarbose (**b**). The data were calculated from three parallel experiments.

Diabetes mellitus is a chronic metabolic disease characterized by hyperglycemia. Studies have shown that α-glucosidase is vital in glucose suitable for intestinal absorption. Consequently, the inhibition of α-glucosidase can significantly assist individuals with diabetes mellitus in maintaining tighter control over their blood glucose levels [71]. In this study, we assessed the anti-α-glucosidase activity of the essential oil. The findings, as presented in Table 3, revealed an IC_50_ value of 174.70 ± 13.12 µg/mL for our essential oil, significantly exceeding the activities of essential oils from *C. sativum* (6.24 ± 0.86 mg/mL) and *C. carvi* (6.83 ± 0.76 mg/mL) [68], suggesting the essential oil exhibits a relatively stronger inhibitory effect against α-glucosidase, which may contribute to the management of diabetes mellitus. Prior research has demonstrated that essential oils from sesquiterpene-rich plants, such as *P. nissolii*, exhibit notable anti-α-glucosidase activity [72,73]. Possessing this characteristic, essential oil encompasses a diverse array of 10 sesquiterpenoid species totaling 9.70%. Notably, hexahydrofarnesyl acetone (4.44%) and caryophyllene oxide (2.69%) are postulated to be the primary constituents within our essential oil that exert the anti-α-glucosidase effect (Figure 4).

### 2.5. Anti-β-Lactamase Activity

**Figure 5:**
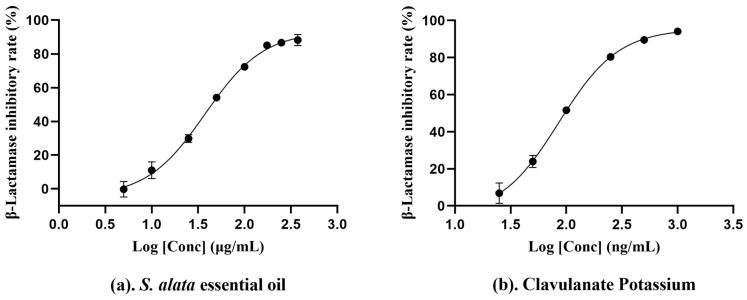
The concentration−dependent anti−β−lactamase activity of *S. alata* essential oil (**a**) and Clavulanate Potassium (**b**). The data were calculated from three parallel experiments.

β-Lactamase, which hydrolyzes β-lactam antibiotics, poses a significant threat to the efficacy of antibacterials. However, drug discovery and development have led to the introduction of β-lactamase inhibitors, providing a novel strategy to surmount this significant clinical challenge [74]. In the experiment, we evaluated the anti-β-lactamase activity of essential oil. The IC_50_ value in Table 3 was 37.56 ± 3.48 µg/mL, indicating that the essential oil had significantly robust anti-β-lactamase activity. However, the record of anti-β-lactamase activity in vitro is currently unavailable. From previous evaluations of the antibacterial activity of bacteria that can produce β-lactamase, we know that monoterpenoids such as citral, laurene, menthol, and camphor have good antibacterial activity. Therefore, we speculate that the abundant terpenoids in the essential oil could be instrumental in its pronounced anti-β-lactamase activity [75,76]. In addition, phenylheptatriyne showed significant selective antibacterial activity against gram-positive bacteria in vitro [49]. Within the composition of this essential oil, phenylheptatriyne (8.07%) is identified as the third most prevalent compound, which may be the primary factor contributing to the essential oil’s potent resistance to β-lactamase (Figure 5).

## 3. Materials and Methods

### 3.1. Plant Materials

The sample of the *S. alata* was collected from Pingnan County (23°16′23.78″ N, 110°30′41.92″ E), Guigang City, the Guangxi Zhuang Autonomous Region, China. After being identified by Professor Hong Zhao, the sample was deposited in the Center for Bioscience Analysis and Testing, Shandong University, Weihai, China. The registration number is EO2304.

### 3.2. Essential Oil Hydrodistillation

The dried leaves and stems (1 kg) were crushed into powder by a grinder and then put into a 5 L round-bottom flask with 2.0 L ultrapure water (Milli-Q Reference, Millipore, Billerica, MA, USA). The essential oil was extracted from the plant materials by hydrodistillation in a Clevenger-type apparatus for about 4 h. The essential oil was separated from the water layer using ether, and the resulting essential oil was then dried by nitrogen (Termovap sample concentrator, MD200-1, Shanghai Huyi Technology Co., Ltd, Shanghai, China) and anhydrous sodium sulfate to obtain the essential oil. The obtained essential oil was stored at a low temperature (−4 °C) for further analysis.

### 3.3. GC-MS and GC-FID Analysis

Agilent gas chromatographic-mass spectrometer (7890-5975C, Agilent, Santa Clara, CA, USA) was used for the GC-MS analysis of the essential oil, equipped with HP-5MS type fused quartz string (30 m × 0.25 mm × 0.25 µm, Agilent, Santa Clara, CA, USA). The gas chromatographic conditions were set as follows: Interface temperature: 280 °C; Injector temperature: 260 °C; Carrier gas: He; Flow rate: 1.0 mL/min; Heating program setting: initial temperature of 50 °C for 4 min, 6 °C/min to 280 °C, and held for 3 min. The mass spectrum conditions were as follows: EI: 70 eV; Scanning range: 25–500 amu; Scan rate: 4.0 scan/s; Quadrupole temperature: 150 °C; Sample size: 0.3 µL [77]. GC-FID analysis was performed using a PerkinElmer gas chromatograph (Clarus 500, Shelton, CT, USA) with an HP-5 fused silica capillary column (30 m × 0.25 mm, film thickness of 0.25 μm, Agilent, Santa Clara, CA, USA). The injector temperature was 260 °C, and the detector temperature was 305 °C. The oven temperature was initially set at 50 °C and held for 4 min, then raised from 50 °C to 280 °C at a rate of 6 °C/min, and maintained steady for 3 min. Nitrogen was used as the carrier gas at a flow rate of 1.1 mL/min [78]. Identifying these compounds in the essential oil was based on comparing mass spectrometry data with the NIST/EPA/NIH 2020 Mass Spectral Database and Kovat’s retention indices associated with retention times. Kovat’s retention indices were calculated by the retention time of a series of *n*-alkanes (C_8_–C_30_) [79].

### 3.4. Antioxidant Capacity Evaluation

#### 3.4.1. DPPH Method

The DPPH radical-scavenging abilities of essential oil were determined according to the procedure in previous studies [80,81]. 6-Hydroxy-2,5,7,8-tetramethylchrome-Roman-2-carboxylic acid (Trolox) was used as the positive control. An amount of 200 µL prepared 0.17 mmol/L DPPH (2,2-diphenyl-1-picroyl-hydrazine hydrate) solution with 50 µL ethanol was added to the microplate as a control. The sample blank was prepared with 50 µL ethanolic essential oil solution and 200 µL ethanol. Then, 50 µL ethanolic essential oil solutions (50, 25, 10, 5, 2.5, and 1 mg/mL) were added to 200 µL DPPH solution in a microplate. After incubating in darkness for 30 min, the microplate reader (Epoch, Biotech company, Minneapolis, MN, USA) was used to measure the absorbance at 516 nm. Microplate Manager software Gen5 (Version 2.09) was used to record the reading of each sample. The absorbance was tested three times to obtain the mean value. Finally, the free radical-scavenging activity (RSA%) was calculated according to the following formula:

$$\mathrm{RSC}\%=\left(1-\frac{A_{\mathrm{Sample}}-A_{\mathrm{Sample}\;\mathrm{Blank}}}{A_{\mathrm{Control}}}\right)\times 100\%$$

where, A_Sample_ is the absorbance of the sample under different concentrations, A_Control_ is the absorbance of the ethanol solution containing DPPH, and A_Sample Blank_ is the absorbance of the ethanol solution without DPPH.

#### 3.4.2. ABTS Method

In the experiment, 2,2-azolium-(3-ethylbenzothiazole-6-sulfonic acid) diammonium salt (ABTS, 7.4 mmol/L) was mixed with potassium persulfate (K_2_S_2_O_8_, 2.6 mmol/L) to produce ABTS^•+^ free radicals. The mixture is placed in a dark environment for 12 h to ensure a full reaction. The resulting ABTS^•+^ was diluted in anhydrous ethanol to obtain the working solution with an absorbance of 0.7 at 34 nm. Then, 200 µL diluted ABTS^•+^ solution was mixed with 50 µL gradient-diluted ethanol solutions (50, 25, 10, 5, 2.5, and 1 mg/mL) in a 96-well microplate. An amount of 50 µL ethanolic essential oil solution and 200 µL ethanol were mixed as a sample blank. After incubation for 6 min, the absorbance was measured at 734 nm [81,82]. The experiment was repeated three times. The inhibition percentage (inhibition%) of the measured essential oil is calculated as follows:

$$I\mathrm{nhibition}\%=\frac{A_{0}-A}{A_{0}}\times 100\%$$

where A_0_ represents the absorbance of 200 μL ABTS^•+^ diluted solution mixed with 50 μL ethanol, and A represents the absorbance of 200 μL ABTS^•+^ diluted solution mixed with 50 μL sample solution. 

#### 3.4.3. FRAP Method

A standard solution of Trolox was used as the positive control, and the blank sample was prepared with distilled water. To obtain the FRAP working reagent, 0.3 M pH 3.6 acetic acid buffer solution, 10 mmol/L TPTZ solution, and 20 mmol/L Fe (III) solution were mixed at the ratio of 10:1:1. 50 µL diluted essential oil solution (5000, 2500, 1000, 500, 250, 100, 50, and 25 µg/mL) and 0.25 mg/mL Trolox solution (2.5, 5, 10, 15, 20, 25, and 50 μL) were mixed with 200 µL FRAP working reagent in a 96-well microplate, followed by incubation in a water bath at 37 °C for 40 min. After 40 min, the absorbance of the tested sample at 593 nm was measured using the microplate reader (Epoch, Biotech company, Minneapolis, MN, USA). All tests were performed in triplicate. The standard curve was constructed using Trolox, and the FRAP value was determined using Trolox as the standard. The absorbance values of the samples at known concentrations are substituted into the standard curve to obtain the equivalent value of Trolox, which serves as the standard for antioxidant capacity [81,83].

### 3.5. Anti-Acetylcholinesterase Activity Test

Ethanolic essential oil solution was diluted with pH 8.0 phosphate-buffered saline (PBS) solution to 2.5, 1.0, 0.5, 0.25, 0.10, and 0.05 mg/mL. Galantamine was used as a positive control. An amount of 145 μL PBS solution (0.1 M, pH = 8.0), 20 μL test sample solution, and 15 μL 0.11 U/mL acetylcholinesterase solution were mixed and then stored at 4 °C for 20 min. After that, 10 μL 2 mM 5,5-dithiobis-(2-nitrobenzoic acid) DTNB and 10 μL 15 mM acetylthiocholine iodide (ATCI) were added. The absorbance of each solution at 412 nm was measured every 1 min for 6 min [10]. The experiments were performed in triplicate. The acetylcholinesterase inhibitory rate was calculated as

$$\mathrm{Inhibition}\%=\frac{K_{E}-K_{S}}{K_{E}}\times 100\%$$

where K_E_ is the initial reaction rate of inhibited acetylcholinesterase, and K_S_ is the initial reaction rate of uninhibited acetylcholinesterase. IC_50_ was calculated using nonlinear regression.

### 3.6. Anti-α-Glucosidase Capacity Test

Ethanolic essential oil solution was diluted with pH 7.0 PBS solution to 5.0, 2.5, 1, 0.5, 0.25, 0.1, 0.05, and 0.025 mg/mL. Acarbose solution was used as the positive control. An amount of 80 μL 100 mM pH 6.8 PBS solution, 20 μL test sample solution, and 40 μL 0.25 U/mL α-glucosidase solution were mixed in a microplate and incubated at 30 °C for 10 min. Then, 20 μL 3.0 mg/mL of 4-nitrophenyl-α-D-glucopyranoside (pNPG) solution was added, homogenized, and incubated for 4 min. The absorbance was measured at 410 nm and recorded every 1 min for 6 min [84]. All tests were performed in triplicate. The α-glucosidase inhibitory rate was calculated as

$$\mathrm{Inhibition}\%=\frac{K_{E}-K_{S}}{K_{E}}\times 100\%$$

where K_E_ is the initial reaction rate of the uninhibited enzyme, and K_S_ is the initial reaction rate of the inhibited enzyme. The IC_50_ value was calculated using nonlinear regression.

### 3.7. Anti-β-Lactamase Capacity Test

Ethanolic essential oil solution was diluted with pH 7.0 PBS solution. Clavulanate Potassium solution was used as the positive control. An amount of 20 μL Test sample solution, 100 μL 1000 U/mL β-lactamase solution, and 30 μL PBS solution (50 mM, pH = 7.0) were added to the microplate. The mixture is incubated at 30 °C for 10 min. Then, 50 μL of Nitrocefin (0.1 mg/mL) was added, and the mixture was further incubated at 30 °C for another 10 min [85,86]. The absorbance was measured at 489 nm. The tests were carried out in triplicate. The β-lactamase inhibitory rate is shown below:

$$\mathrm{Inhibition}\%=\left(1-\frac{A_{s}-A_{\mathrm{sb}}}{A_{e}-A_{b}}\right)\times 100\%$$

where A_S_ is the absorbance of the sample containing essential oil, A_sb_ is the blank reaction absorbance of the sample, A_e_ is the enzymatic determination absorbance, and A_b_ is the blank reaction absorbance. The IC_50_ value was calculated using nonlinear regression.

## 4. Conclusions

The present study found that the essential oil of *S. alata* is a fatty acid chemotype containing major volatile compounds such as palmitic acid, linoleic acid, phenylheptatriyne, hexahydrofernesyl acetone, and tetradecanoic acid. The essential oil demonstrated moderate DPPH radical-scavenging ability (IC_50_ > 10 mg/mL), ABTS^•+^ radical-scavenging ability (IC_50_ = 3.84 ± 2.12 mg/mL), and FRAP total antioxidant capacity (87.22 ± 12.22 µM/g). In addition, the essential oil showed notable acetylcholinesterase inhibitory activity, with an IC_50_ value of 286.0 ± 79.04 μg/mL. This study also revealed significant anti-α-glucosidase and anti-β-lactamase activities (IC_50_ = 174.7 ± 13.12 μg/mL and 37.56 ± 3.48 μg/mL, respectively). Our results suggest that the essential oil may possess medicinal value in treating diseases such as Alzheimer’s disease and diabetes mellitus. Additionally, it could be used in combination with antibiotics to enhance their antibacterial effects. However, further research involving in silico analysis, such as molecular docking and molecular dynamics simulations, as well as in vivo experiments, is necessary for the development and application of *S. alata* essential oil in the future.

## Figures and Tables

**Table 1:** Chemical composition of essential oil distilled from *S. alata.*

No.	RT	Compound	RI^calc^	RI^lib^	Area (%)	Identification Method	CAS ID
1	10.688	Linalool oxide	1076	1074	0.78%	RRI, MS	5989-33-3
2	11.441	Linalool	1104	1099	3.08%	RRI, MS	78-70-6
3	12.794	(*E*,*Z*)-2,6-Nonadienal	1157	1155	0.24%	RRI, MS	557-48-2
4	12.952	(*E*)-2-Nonenal	1163	1162	0.37%	RRI, MS	18829-56-6
5	13.110	Camphol	1169	1167	0.56%	RRI, MS	507-70-0
6	13.285	1-Nonanol	1176	1173	0.17%	RRI, MS	143-08-8
7	13.732	α-Terpineol	1193	1189	0.39%	RRI, MS	98-55-5
8	14.087	Decanal	1208	1206	0.36%	RRI, MS	112-31-2
9	14.447	β-Cyclocitral	1223	1220	0.17%	RRI, MS	432-25-7
10	14.621	Nerol	1230	1228	0.33%	RRI, MS	106-25-2
11	15.309	Geraniol	1260	1255	0.28%	RRI, MS	106-24-1
12	15.418	(*E*)-2-Decenal	1264	1263	0.62%	RRI, MS	3913-81-3
13	15.974	Isobornyl acetate	1288	1286	0.27%	RRI, MS	125-12-2
14	16.165	(*E*,*Z*)-2,4-Decadienal	1296	1295	0.20%	RRI, MS	25152-83-4
15	16.689	(*E*,*E*)-2,4-Decadienal	1319	1317	0.51%	RRI, MS	25152-84-5
16	16.885	4-Ethyl-2-methoxyanisole	1328	1320	0.26%	RRI, MS	5888-51-7
17	17.725	2-Undecenal	1366	1367	0.35%	RRI, MS	2463-77-6
18	18.085	*n*-Decanoic acid	1382	1372	0.23%	RRI, MS	334-48-5
19	18.211	Damascenone	1388	1386	1.19%	RRI, MS	23726-93-4
20	18.615	Hexahydropseudoionone	1407	1406	0.64%	RRI, MS	1604-34-8
21	18.926	Caryophyllene	1422	1419	0.19%	RRI, MS	87-44-5
22	19.122	β-Copaene	1431	1432	0.31%	RRI, MS	18252-44-3
23	19.215	Mellitene	1436	1434	0.14%	RRI, MS	87-85-4
24	19.635	Dihydropseudoionone	1456	1452	0.78%	RRI, MS	689-67-8
25	19.755	4-Methyl-tetradecane	1462	1459	0.15%	RRI, MS	25117-24-2
26	19.858	Precocene I	1467	1466	0.80%	RRI, MS	17598-02-6
27	20.071	Undecanoic acid	1477	1468	0.17%	RRI, MS	112-37-8
28	20.333	(*E*)-β-Ionone	1490	1486	1.29%	RRI, MS	79-77-6
29	21.026	4-(2-Methyl-3-oxocyclohexyl)-butanal	1525	1515	0.27%	RRI, MS	92485-93-3
30	21.140	3-(2-Pentenyl)-1,2,4-cyclopentanetrione	1530	1525	0.39%	RRI, MS	54644-27-8
31	21.866	(*E*)-Nerolidol	1567	1564	0.09%	RRI, MS	7212-44-4
32	22.073	Dodecanoic acid	1578	1568	0.52%	RRI, MS	143-07-7
33	22.188	(-)-Spathulenol	1584	1577	0.42%	RRI, MS	77171-55-2
34	22.292	Caryophyllene oxide	1589	1581	2.69%	RRI, MS	1139-30-6
35	22.482	Mintketone	1599	1595	0.53%	RRI, MS	73809-82-2
36	22.777	Humulene oxide II	1615	1606	0.40%	RRI, MS	19888-34-7
37	23.039	Silphiperfol-6-en-5-one	1629	1623	0.13%	RRI, MS	77887-60-6
38	23.159	Isospathulenol	1635	1638	0.16%	RRI, MS	88395-46-4
39	23.284	5-Heptene-1,3-diynylbenzene	1642	1642	0.96%	RRI, MS	13678-98-3
40	23.628	α-Cadinol	1660	1653	0.17%	RRI, MS	481-34-5
41	23.715	Precocene II	1665	1558	0.59%	RRI, MS	644-06-4
42	23.912	(*E*)-2-Tetradecenal	1676	1673	0.38%	RRI, MS	51534-36-2
43	23.961	1-Tetradecanol	1678	1676	0.51%	RRI, MS	112-72-1
44	24.354	Heptadecane	1699	1700	0.14%	RRI, MS	629-78-7
45	24.659	Pentadecanal	1717	1717	0.68%	RRI, MS	2765-11-9
46	24.921	Phenylheptatriyne	1731	1725	8.07%	RRI, MS	4300-27-0
47	25.739	Tetradecanoic acid	1778	1768	3.16%	RRI, MS	544-63-8
48	26.579	Methyl pentadecanoate	1827	1824	0.14%	RRI, MS	7132-64-1
49	26.950	Hexahydrofarnesyl acetone	1849	1844	4.44%	RRI, MS	502-69-2
50	27.365	Pentadecanoic acid	1873	1867	1.70%	RRI, MS	1002-84-2
51	27.649	(*Z*,*Z*)-8,11-Heptadecadienal	1890	1886	0.48%	RRI, MS	56797-42-3
52	27.752	Methyl (4*E*,7*E*,10*E*)-4,7,10-hexadecatrienoate	1896	1892	0.29%	RRI, MS	17364-31-7
53	28.172	Farnesyl acetone	1922	1919	0.30%	RRI, MS	1117-52-8
54	28.260	Methyl palmitate	1927	1926	0.51%	RRI, MS	112-39-0
55	28.614	Palmitoleic acid	1949	1951	1.35%	RRI, MS	373-49-9
56	29.324	Palmitic acid	1964	1968	30.74%	RRI, MS	57-10-3
57	30.213	Cycloheptadecanolide	2051	2042	0.15%	RRI, MS	5637-97-8
58	30.524	Heptadecanoic acid	2071	2071	0.15%	RRI, MS	506-12-7
59	30.911	Methyl linoleate	2096	2092	0.28%	RRI, MS	112-63-0
60	31.020	Methyl linolenate	2103	2098	0.51%	RRI, MS	301-00-8
61	31.080	γ-Hexadecalactone	2107	2105	0.35%	RRI, MS	730-46-1
62	31.206	Phytol	2116	2114	1.25%	RRI, MS	150-86-7
63	31.882	Linolenic acid	2130	2139	16.13%	RRI, MS	60-33-3
64	32.068	Octadecanoic acid	2174	2172	0.88%	RRI, MS	57-11-4
65	32.297	Hexadecanamide	2190	2184	0.21%	RRI, MS	629-54-9
66	33.835	Tricosane	2298	2300	0.24%	RRI, MS	638-67-5
67	34.626	4,8,12,16-Tetramethylheptadecan-4-olide	2357	2364	0.25%	RRI, MS	96168-15-9
Fatty acids					55.03%		
Esters					2.31%		
Monoterpenoids					9.49%		
Sesquiterpenoids					9.70%		
Diterpenoids					1.25%		
Aldehydes (including aldehydes and olefine aldehyde)					4.75%		
Aromatic compounds					9.43%		
Other compounds					3.48%		
Total identified					95.44%		

Concentration is calculated from the total ion chromatogram; RI^Calc^: Calculated retention index. RI^lib^: Retention index obtained from the mass spectral database. RRI: Relative retention indices calculated against n-alkanes; Identification method based on the relative retention indices (RRI) of authentic compounds on the HP-5MS column; MS, identified based on computer matching of the mass spectra with NIST/EPA/NIH 2020 Mass Spectral Database and comparison with literature data.

**Table 2:** Antioxidant activities expressed as IC_50_ values for DPPH, ABTS, and the antioxidant capacity of FRAP assays.

Tested Samples	DPPH (IC_50_)	ABTS (IC_50_)	FRAP Antioxidant Capacity
*S. alata* essential oil	>10 mg/mL	3.84 ± 2.12 mg/mL	87.22 ± 12.22 µM/g
Trolox	9.3 ± 1.3 µg/mL	6.1 ± 1.4 µg/mL	-

**Table 3:** Enzyme inhibitory activities expressed as IC_50_ values for anti-acetylcholinesterase, anti-α-glucosidase, and anti-β-lactamase assays.

Tested Samples	Anti-Acetylcholinesterase (IC_50_)	Anti-α-Glucosidase (IC_50_)	Anti-β-Lactamase (IC_50_)
*S. alata* essential oil	286.0 ± 79.04 μg/mL	174.7 ± 13.12 μg/mL	37.56 ± 3.48 μg/mL
Galantamine	130.0 ± 2.0 ng/mL	-	-
Acarbose	-	6.40 ± 0.46 ng/mL	-
Clavulanate Potassium	-	-	85.98 ± 10.37 ng/mL

## Data Availability

The data presented in this study are available on request from the corresponding author.

## Supplementary Materials

The following supporting information can be downloaded at: https://www.mdpi.com/article/10.3390/molecules29122869/s1.
