# Supplementary material for: Spermacoce alata Aubl. Essential Oil: Chemical Composition, In Vitro Antioxidant Activity, and Inhibitory Effects of Acetylcholinesterase, α-Glucosidase and β-Lactamase
Source: Molecules. 2024 Jun 16;29(12):2869. doi: 10.3390/molecules29122869 (PMC11206966; doi:10.3390/molecules29122869)
Supplement: Supplementary file 1 [file molecules-29-02869-s001.zip › EO2304 GC-FID.pdf]

chromatogram

sample name: sample #:

file name: F:\liuxu\20231122\EO2304.raw

date: 2023-11-30 20:29:15

method: 2023-02 injection time: 2023-11-23 15:52:14

start time: 4.00 min end time: 45.33 min low point: 0.00 mV high point.........mV

Graph offset: 0.00 mV graph scale ..mV

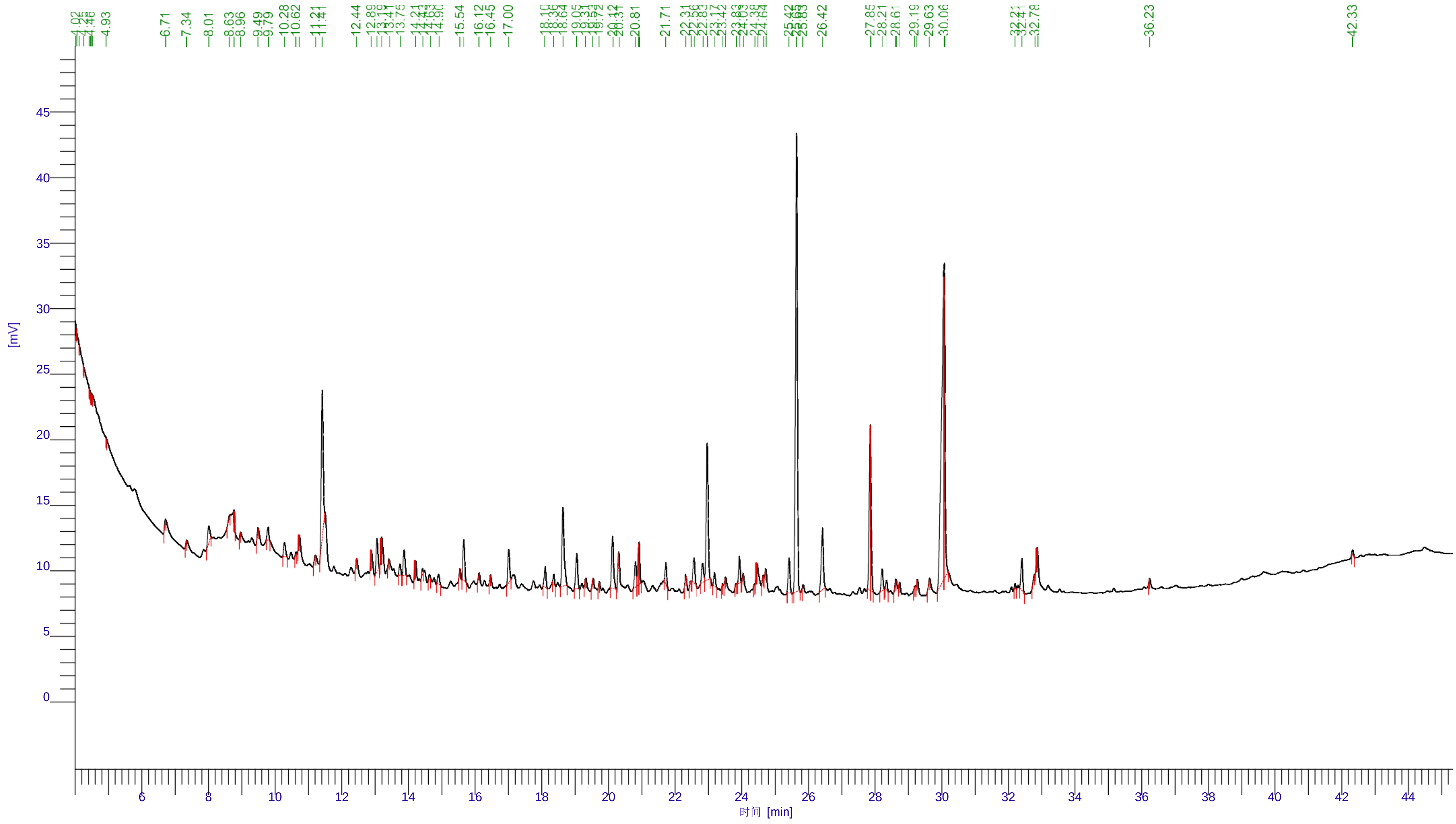

software version : 6.3.2.0646
handlers : manager
sample number :
autosampler : NONE
Instrument name : Clarus500
Instrument series # : 650N7112101
delay time : 4.00 min
sample rate: 25.0000
injection volume: 1.000000ul

date : 2009-3-10 20:18:39
sample name:
research :
Bottle position: 0/0
channel : A
A/D mV range: 1000
end time : 45.33 min
area exclusion: 0.000000dilution
factor: 1.00 circulation: 1

Sample content : 1.0000
Data acquisition time: 2023-11-23 15:52:14
raw data file: F:\liuxu\20231122\EO2304.raw
destination file: f:\liuxu\20231122\eo2304.rst
instrumental method: F:\method\2023-02 from F:\liuxu\20231122\EO2304.raw
handling method: F:\method\2023-02 from f:\liuxu\20231122\eo2304.rst
calibration method: F:\method\2023-02 from f:\liuxu\20231122\eo2304.rst
report format file: F:\method\2023-11.rpt
sequential file: C:\PenExe\TcWS\Ver6.3.2\Examples\EO2304.seq

DEFAULT REPORT

peak #	time [min]	area [µV·s]	height [µV]	area [%]	normalized area [%]	BL	area/height [s]
1	4.015	284.31	361.73	0.04	0.04	BB	0.7860
2	4.043	120.61	239.33	0.02	0.02	BB	0.5039
3	4.119	46.58	111.42	0.01	0.01	BB	0.4180
4	4.250	127.14	248.46	0.02	0.02	BB	0.5117
5	4.421	57.63	107.71	0.01	0.01	BB	0.5350
6	4.459	80.05	134.33	0.01	0.01	BB	0.5959
7	4.481	58.88	106.26	0.01	0.01	BB	0.5541
8	4.517	84.10	146.78	0.01	0.01	BB	0.5730
9	4.926	106.41	143.21	0.02	0.02	BB	0.7430
10	6.708	726.12	220.75	0.11	0.11	BB	3.2893
11	7.342	208.48	80.23	0.03	0.03	BB	2.5987
12	8.009	5585.14	1274.76	0.81	0.81	BB	4.3813
13	8.631	669.75	164.99	0.10	0.10	BB	4.0593
14	8.771	1410.89	834.31	0.21	0.21	BB	1.6911
15	8.959	229.25	85.72	0.03	0.03	BB	2.6743
16	9.488	648.24	239.53	0.09	0.09	BB	2.7063
17	9.792	3490.54	997.90	0.51	0.51	BB	3.4979
18	10.277	4419.22	1063.58	0.64	0.64	BB	4.1551
19	10.622	582.22	255.15	0.08	0.08	BB	2.2819
20	10.723	546.42	187.28	0.08	0.08	BB	2.9177
21	11.211	581.82	74.11	0.08	0.08	BB	7.8509
22	11.410	44145.85	11277.46	6.44	6.44	BB	3.9145
23	12.441	278.49	103.32	0.04	0.04	BB	2.6952
24	12.888	390.19	138.80	0.06	0.06	BB	2.8112
25	13.053	9202.64	2535.32	1.34	1.34	BB	3.6298
26	13.193	1458.02	460.77	0.21	0.21	BB	3.1643
27	13.412	299.79	159.87	0.04	0.04	BB	1.8752
28	13.746	2916.13	851.29	0.43	0.43	BB	3.4255
29	13.869	7245.82	1915.78	1.06	1.06	BB	3.7822
30	14.207	457.17	169.16	0.07	0.07	BB	2.7026
31	14.426	1139.58	357.90	0.17	0.17	BB	3.1841

peak #	time [min]	area [µV·s]	height [µV]	area [%]	normalized area [%]	BL	area/height [s]
32	14.633	993.47	362.19	0.14	0.14	BB	2.7429
33	14.899	2833.74	712.81	0.41	0.41	BB	3.9754
34	15.544	232.22	106.86	0.03	0.03	BB	2.1730
35	15.660	11338.31	3117.87	1.65	1.65	BB	3.6365
36	16.116	283.04	98.86	0.04	0.04	BB	2.8631
37	16.453	168.10	81.40	0.02	0.02	BB	2.0651
38	17.003	9571.49	2562.16	1.40	1.40	BB	3.7357
39	18.096	5597.47	1599.72	0.82	0.82	BB	3.4990
40	18.355	1973.53	707.01	0.29	0.29	BB	2.7914
41	18.635	27372.10	5981.60	3.99	3.99	BB	4.5761
42	19.048	11083.43	2688.95	1.62	1.62	BB	4.1218
43	19.311	210.15	76.99	0.03	0.03	BB	2.7296
44	19.533	385.32	80.80	0.06	0.06	BB	4.7691
45	19.720	216.26	87.59	0.03	0.03	BB	2.4689
46	20.122	16565.81	3984.12	2.42	2.42	BB	4.1580
47	20.306	839.38	277.58	0.12	0.12	BB	3.0240
48	20.810	7785.83	1935.03	1.14	1.14	BV	4.0236
49	20.907	6305.79	3128.14	0.92	0.92	VV	2.0158
50	20.919	6802.00	3214.62	0.99	0.99	VB	2.1160
51	21.714	4812.71	1574.10	0.70	0.70	BB	3.0574
52	22.308	468.70	296.73	0.07	0.07	BB	1.5795
53	22.465	369.29	64.78	0.05	0.05	BB	5.7007
54	22.562	7337.44	1991.75	1.07	1.07	BB	3.6839
55	22.827	6164.76	1476.82	0.90	0.90	BV	4.1743
56	22.963	42426.03	10456.72	6.19	6.19	VB	4.0573
57	23.175	3447.88	1053.48	0.50	0.50	BB	3.2728
58	23.416	117.71	77.83	0.02	0.02	BB	1.5124
59	23.504	235.00	108.52	0.03	0.03	BB	2.1655
60	23.831	117.65	47.40	0.02	0.02	BB	2.4823
61	23.929	6127.65	2024.22	0.89	0.89	BB	3.0272
62	24.034	318.79	170.06	0.05	0.05	BB	1.8746
63	24.379	147.28	80.08	0.02	0.02	BB	1.8392
64	24.459	592.94	125.44	0.09	0.09	BB	4.7268
65	24.635	429.25	147.33	0.06	0.06	BB	2.9135
66	24.705	194.82	164.29	0.03	0.03	BB	1.1859
67	25.422	10114.54	2660.22	1.48	1.48	BB	3.8022
68	25.647	136415.47	34964.85	19.90	19.90	BB	3.9015
69	25.832	85.91	61.94	0.01	0.01	BB	1.3870
70	26.422	20025.87	4699.59	2.92	2.92	BB	4.2612
71	27.852	25976.94	12572.99	3.79	3.79	BV	2.0661
72	27.858	24954.47	12639.65	3.64	3.64	VB	1.9743
73	28.205	5917.86	1668.06	0.86	0.86	BB	3.5477
74	28.338	2003.22	679.17	0.29	0.29	BB	2.9495
75	28.615	634.56	237.09	0.09	0.09	BB	2.6765
76	28.722	66.38	59.33	0.01	0.01	BB	1.1189
77	29.188	175.28	48.65	0.03	0.03	BB	3.6031
78	29.255	110.65	121.90	0.02	0.02	BB	0.9077
79	29.627	832.92	265.86	0.12	0.12	BB	3.1329
80	30.058	111964.52	23615.20	16.33	16.33	BV	4.7412
81	30.078	61930.50	24069.68	9.03	9.03	VB	2.5730
82	32.206	886.48	363.37	0.13	0.13	BB	2.4396
83	32.409	9128.15	2451.11	1.33	1.33	BB	3.7241

peak #	time [min]	area [µV·s]	height [µV]	area [%]	normalized area [%]	BL	area/height [s]
84	32.781	711.12	101.72	0.10	0.10	BB	6.9912
85	32.863	1077.09	438.19	0.16	0.16	BB	2.4580
86	36.228	109.26	88.44	0.02	0.02	BB	1.2354
87	42.330	1073.88	386.66	0.16	0.16	BB	2.7773
		685657.91	197904.77	100.00	100.00		
