# Supplementary material for: Spermacoce alata Aubl. Essential Oil: Chemical Composition, In Vitro Antioxidant Activity, and Inhibitory Effects of Acetylcholinesterase, α-Glucosidase and β-Lactamase
Source: Molecules. 2024 Jun 16;29(12):2869. doi: 10.3390/molecules29122869 (PMC11206966; doi:10.3390/molecules29122869)
Supplement: Supplementary file 1 [file molecules-29-02869-s001.zip › EO2304 GC-MS analysis report .pdf]

Analysis Report

Sample Information

SampleName	Sample 5	DataFilePath	F:\E02304.D
SampleID		AcqTime_Local	2023/12/4 19:03:27 (UTC+08:00)
InstrumentName	GCMS	MethodPath_Acquisition	D:\MassHunter\GCMS\1\methods\2023\2023sample-2.M
MSType	Q	Version_AcqSW	MassHunter GC/MS Acquisition B.07.06.2704 18-Jul-2017 Copyright © 1989-2017 Agilent Technologies, Inc.
InjectionVolume	0.3	IRMStatus	
SamplePosition	5	MethodPath_Analysis	F:\E02304.D\Results\Qual\Version4\deanwhite 240123.m
PlatePosition		TargetSourcePath	D:\MassHunter\Library\NIST20.L;D:\MassHunter\PCDL\Pesticide_Example.cdb;D:\MassHunter\PCDL\Test_AM_PCDL.cdb
Operator		ResultSummary	

Sample Chromatograms

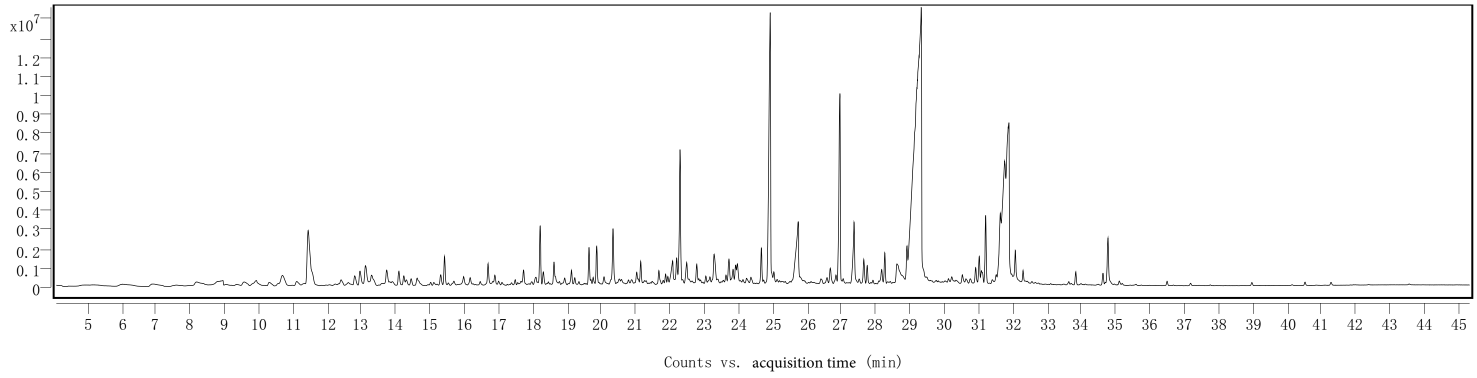

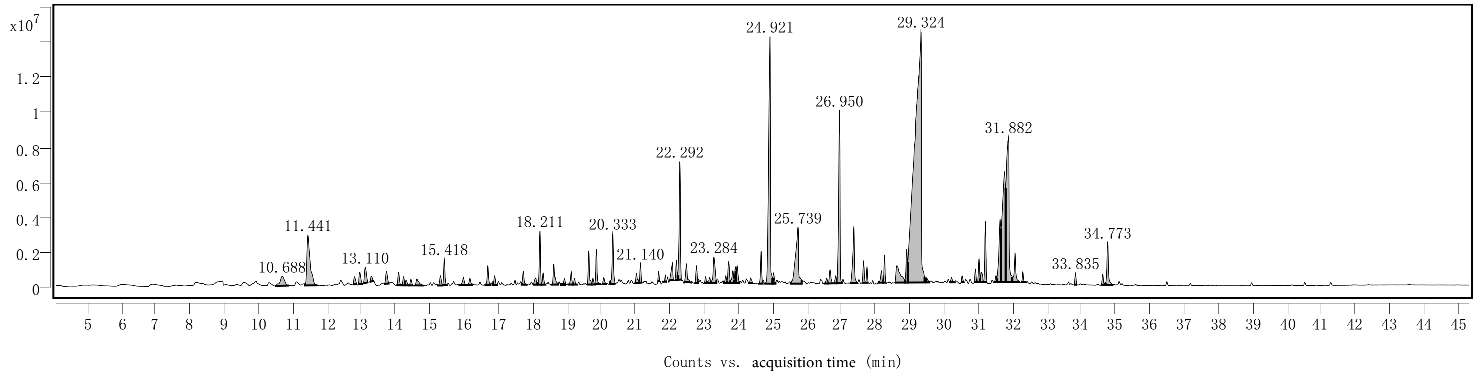

Chromatogram Peaks

Peak	StartRT	RT	EndRT	Height	Area	AreaPct	SNR
1	10.471	10.688	10.894	537112	4855163	2.53	
2	11.359	11.441	11.719	2866461	19276450	10.03	
3	12.757	12.794	12.867	432610	1518455	0.79	
4	12.911	12.952	13.039	656799	2304301	1.20	
5	13.056	13.110	13.197	887866	3502895	1.82	
6	13.240	13.285	13.361	306699	1031500	0.54	
7	13.659	13.732	13.792	698322	2431626	1.27	
8	14.021	14.087	14.179	729098	2275412	1.18	
9	14.179	14.229	14.272	491627	1493767	0.78	
10	14.272	14.299	14.381	282380	1040740	0.54	
11	14.381	14.447	14.505	342050	1077006	0.56	
12	14.545	14.621	14.801	380603	2046639	1.07	
13	15.243	15.309	15.363	555053	1755606	0.91	
14	15.363	15.418	15.494	1490750	3861636	2.01	
15	15.860	15.974	16.083	436091	1710483	0.89	
16	16.084	16.165	16.225	381383	1252871	0.65	
17	16.618	16.689	16.765	1106564	3172546	1.65	
18	16.847	16.885	16.956	516934	1608857	0.84	
19	17.676	17.725	17.818	751370	2189546	1.14	
20	18.009	18.085	18.118	379658	1423434	0.74	
21	18.151	18.211	18.255	3070675	7463590	3.89	
22	18.255	18.304	18.369	660098	1808792	0.94	
23	18.549	18.615	18.735	1163094	3968518	2.07	
24	18.806	18.926	18.980	334406	1200004	0.62	
25	19.046	19.122	19.166	732539	1945621	1.01	
26	19.166	19.215	19.286	349235	878405	0.46	
27	19.586	19.635	19.722	1930526	4853899	2.53	
28	19.722	19.755	19.809	367409	938084	0.49	
29	19.809	19.858	19.995	1968244	4995267	2.60	
30	20.028	20.071	20.162	358355	1037400	0.54	
31	20.262	20.333	20.411	2871652	8056889	4.19	
32	20.971	21.026	21.102	550654	1701276	0.89	
33	21.102	21.140	21.183	1092086	2436816	1.27	
34	21.644	21.670	21.715	533347	1054111	0.55	
35	21.844	21.866	21.893	358937	587048	0.31	
36	21.990	22.073	22.117	981337	3226231	1.68	
37	22.146	22.188	22.226	1109130	2652369	1.38	
38	22.226	22.292	22.331	6752706	16782508	8.74	

Chromatogram Peaks

Peak	StartRT	RT	EndRT	Height	Area	AreaPct	SNR
39	22.430	22.482	22.608	987845	3289771	1.71	
40	22.714	22.777	22.815	962582	2482603	1.29	
41	23.006	23.039	23.083	351151	791971	0.41	
42	23.083	23.159	23.197	307721	975265	0.51	
43	23.197	23.284	23.355	1488050	6029838	3.14	
44	23.584	23.628	23.666	404757	1090775	0.57	
45	23.666	23.715	23.759	1223897	3692974	1.92	
46	23.759	23.835	23.874	700530	2448991	1.27	
47	23.874	23.912	23.934	921381	2362840	1.23	
48	23.934	23.961	24.026	988751	3155989	1.64	
49	24.310	24.354	24.414	292870	903886	0.47	
50	24.599	24.659	24.703	1838285	4249845	2.21	
51	24.785	24.921	24.992	14125373	50411223	26.24	
52	24.992	25.025	25.079	603932	1802516	0.94	
53	25.527	25.739	25.843	3216497	19758412	10.29	
54	26.493	26.579	26.618	294326	889523	0.46	
55	26.618	26.672	26.747	785296	2426351	1.26	
56	26.770	26.836	26.874	423802	1282822	0.67	
57	26.874	26.950	27.021	9884696	27765759	14.45	
58	27.268	27.365	27.474	3158060	10643282	5.54	
59	27.602	27.649	27.695	1192163	3001640	1.56	
60	27.715	27.752	27.787	881755	1829743	0.95	
61	28.107	28.172	28.216	668202	1862405	0.97	
62	28.216	28.260	28.298	1538696	3207538	1.67	
63	28.563	28.614	28.854	954969	8443219	4.40	
64	28.854	28.909	28.936	1885034	5167517	2.69	
65	28.936	29.324	29.427	14333072	192101606	100.00	
66	29.465	29.482	29.569	266331	1021086	0.53	
67	30.169	30.213	30.311	270469	956964	0.50	
68	30.493	30.524	30.587	374380	928814	0.48	
69	30.874	30.911	30.964	746406	1759752	0.92	
70	30.973	31.020	31.053	1331621	3210414	1.67	
71	31.053	31.080	31.145	579783	2160189	1.12	
72	31.157	31.206	31.251	3466732	7812550	4.07	
73	31.451	31.517	31.538	372819	979781	0.51	
74	31.538	31.631	31.658	3577824	13939567	7.26	
75	31.658	31.757	31.795	6290425	40062667	20.85	
76	31.795	31.882	31.986	8297383	46800504	24.36	
77	31.991	32.068	32.204	1636815	5508967	2.87	
78	32.251	32.297	32.395	577656	1288187	0.67	
79	33.789	33.835	33.875	667140	1510084	0.79	
80	34.567	34.626	34.664	616453	1560427	0.81	
81	34.713	34.773	34.910	2451463	7937233	4.13	

Compound Summary

Cpd	CpdName	Formula	RT	Mass	CAS	IDSourceBest	Score_ID	Score_Lib	Score_DB	Score_MFG	Algorithm
1	2-Furanmethanol, 5-ethenyltetrahydro-.alpha.,.alpha.,5-trimethyl-, cis-	C10 H18 O2	10.688		5989-33-3	Lib searching	83.26	83.26			integral
2	Linalool	C10 H18 O	11.441		78-70-6	Lib searching	98.60	98.60			integral
3	2,6-Nonadienal, (E,Z)-	C9 H14 O	12.794		557-48-2	Lib searching	90.44	90.44			integral
4	2-Nonenal, (E)-	C9 H16 O	12.952		18829-56-6	Lib searching	94.98	94.98			integral
5	endo-Borneol	C10 H18 O	13.110		507-70-0	Lib searching	96.14	96.14			integral
6	1-Nonanol	C9 H20 O	13.285		143-08-8	Lib searching	100.00				integral
7	.alpha.-Terpineol	C10 H18 O	13.732		98-55-5	Lib searching	92.81	92.81			integral
8	Decanal	C10 H20 O	14.087		112-31-2	Lib searching	91.26	91.26			integral
9	3-Cyclohexene-1-acetaldehyde, .alpha.,4-dimethyl-	C10 H16 O	14.229		29548-14-9	Lib searching	85.31	85.31			integral
10	?		14.299			Lib searching	100.00				integral
11	1-Cyclohexene-1-carboxaldehyde, 2,6,6-trimethyl-	C10 H16 O	14.447		432-25-7	Lib searching	93.53	93.53			integral
12	Nerol	C10 H18 O	14.621		106-25-2	Lib searching	100.00				integral
13	Geraniol	C10 H18 O	15.309		106-24-1	Lib searching	81.85	81.85			integral
14	2-Decenal, (E)-	C10 H18 O	15.418		3913-81-3	Lib searching	84.83	84.83			integral
15	Isobornyl acetate	C12 H20 O2	15.974		125-12-2	Lib searching	71.32	71.32			integral
16	2,4-Decadienal, (E,Z)-	C10 H16 O	16.165		106-25-2	Lib searching	100.00				integral
17	2,4-Decadienal, (E,E)-	C10 H16 O	16.689		25152-84-5	Lib searching	93.35	93.35			integral
18	Benzene, 4-ethyl-1,2-dimethoxy-	C10 H14 O2	16.885		5888-51-7	Lib searching	78.73	78.73			integral
19	2-Undecenal	C11 H20 O	17.725		2463-77-6	Lib searching	86.83	86.83			integral
20	n-Decanoic acid	C10 H20 O2	18.085		334-48-5	Lib searching	79.77	79.77			integral
21	2-Buten-1-one, 1-(2,6,6-trimethyl-1,3-cyclohexadien-1-yl)-, (E)-	C13 H18 O	18.211		23726-93-4	Lib searching	96.45	96.45			integral
22	?		18.304			Lib searching	100.00				integral
23	2-Undecanone, 6,10-dimethyl-	C13 H26 O	18.615		1604-34-8	Lib searching	71.07	71.07			integral
24	Caryophyllene	C15 H24	18.926		87-44-5	Lib searching	84.65	84.65			integral
25	(1R,2S,6S,7S,8S)-8-Isopropyl-1-methyl-3-methylenetricyclo[4.4.0.02,7]decane-rel-	C15 H24	19.122		18252-44-3	Lib searching	89.83	89.83			integral
26	Benzene, hexamethyl-	C12 H18	19.215		87-85-4	Lib searching	100.00				integral
27	5,9-Undecadien-2-one, 6,10-dimethyl-	C13 H22 O	19.635		689-67-8	Lib searching	96.62	96.62			integral
28	Tetradecane, 4-methyl-	C15 H32	19.755		25117-24-2	Lib searching	100.00				integral
29	Precocene I	C12 H14 O2	19.858		17598-02-6	Lib searching	97.78	97.78			integral
30	Undecanoic acid	C11 H22 O2	20.071		112-37-8	Lib searching	100.00				integral
31	trans-.beta.-Ionone	C13 H20 O	20.333		79-77-6	Lib searching	84.42	84.42			integral

Compound Summary

Cpd	CpdName	Formula	RT	Mass	CAS	IDSourceBest	Score_ID	Score_Lib	Score_DB	Score_MFG	Algorithm
32	4-(2-Methyl-3-oxocyclohexyl)butanal	C11 H18 O2	21.026		92485-93-3	Lib searching	100.00				integral
33	1,2,4-Cyclopentanetrione, 3-(2-pentenyl)-	C10 H12 O3	21.140		54644-27-8	Lib searching	100.00				integral
34	?	C15 H26 O	21.670			Lib searching	100.00				integral
35	E-Nerolidol	C15 H26 O	21.866		40716-66-3	Lib searching	100.00				integral
36	Dodecanoic acid	C12 H24 O2	22.073		143-07-7	Lib searching	94.37	94.37			integral
37	(-)-Spathulenol	C15 H24 O	22.188		77171-55-2	Lib searching	100.00				integral
38	Caryophyllene oxide	C15 H24 O	22.292		1139-30-6	Lib searching	97.24	97.24			integral
39	Mintketone	C15 H24 O	22.482		73809-82-2	Lib searching	100.00				integral
40	Humulene oxide II	C15 H24 O	22.777		19888-34-7	Lib searching	100.00				integral
41	Silphiperfol-6-en-5-one	C15 H22 O	23.039		77887-60-6	Lib searching	82.64	82.64			integral
42	Isospathulenol	C15 H24 O	23.159		88395-46-4	Lib searching	84.52	84.52			integral
43	Benzene, 5-heptene-1,3-diyn-1-yl-	C13 H10	23.284		13678-98-3	Lib searching	85.05	85.05			integral
44	.alpha.-Cadinol	C15 H26 O	23.628		481-34-5	Lib searching	84.64	84.64			integral
45	Precocene II	C13 H16 O3	23.715		644-06-4	Lib searching	100.00				integral
46	?		23.835			Lib searching	100.00				integral
47	(E)-Tetradec-2-enal	C14 H26 O	23.912		51534-36-2	Lib searching	100.00				integral
48	1-Tetradecanol	C14 H30 O	23.961		112-72-1	Lib searching	100.00				integral
49	Heptadecane	C17 H36	24.354		629-78-7	Lib searching	83.05	83.05			integral
50	Pentadecanal-	C15 H30 O	24.659		2765-11-9	Lib searching	97.66	97.66			integral
51	Benzene, 1,3,5-heptatriyn-1-yl-	C13 H8	24.921		4300-27-0	Lib searching	97.50	97.50			integral
52	delete		25.025			Lib searching	100.00				integral
53	Tetradecanoic acid	C14 H28 O2	25.739		544-63-8	Lib searching	97.15	97.15			integral
54	Methyl pentadecanoate	C16 H32 O2	26.579		7132-64-1	Lib searching	100.00				integral
55	delete		26.672			Lib searching	100.00				integral
56	?		26.836			Lib searching	100.00				integral
57	2-Pentadecanone, 6,10,14-trimethyl-	C18 H36 O	26.950		502-69-2	Lib searching	97.01	97.01			integral
58	Pentadecanoic acid	C15 H30 O2	27.365		1002-84-2	Lib searching	74.96	74.96			integral
59	8,11-Heptadecadienal, (Z,Z)-		27.649		56797-42-3	Lib searching	100.00				integral
60	4,7,10-Hexadecatrienoic acid, methyl ester	C17 H28 O2	27.752		17364-31-7	Lib searching	100.00				integral
61	5,9,13-Pentadecatrien-2-one, 6,10,14-trimethyl-, (E,E)-	C18 H30 O	28.172		1117-52-8	Lib searching	87.95	87.95			integral
62	Hexadecanoic acid, methyl ester	C17 H34 O2	28.260		112-39-0	Lib searching	96.86	96.86			integral
63	Palmitoleic acid	C16 H30 O2	28.614		373-49-9	Lib searching	93.53	93.53			integral
64	Dibutyl phthalate	C16 H22 O4	28.909		84-74-2	Lib searching	86.87	86.87			integral
65	n-Hexadecanoic acid	C16 H32 O2	29.324		57-10-3	Lib searching	98.51	98.51			integral
66	delete		29.482			Lib searching	100.00				integral
67	Cycloheptadecanolide		30.213		5637-97-8	Lib searching	100.00				integral
68	Heptadecanoic acid	C17 H34 O2	30.524		506-12-7	Lib searching	85.25	85.25			integral
69	9,12-Octadecadienoic acid (Z,Z)-, methyl ester	C19 H34 O2	30.911		112-63-0	Lib searching	94.53	94.53			integral
70	9,12,15-Octadecatrienoic acid, methyl ester, (Z,Z,Z)-	C19 H32 O2	31.020		301-00-8	Lib searching	91.66	91.66			integral
71	2(3H)-Furanone, 5-dodecyldihydro-	C16 H30 O2	31.080		730-46-1	Lib searching	77.52	77.52			integral
72	Phytol	C20 H40 O	31.206		150-86-7	Lib searching	97.98	97.98			integral
73	delete		31.517			Lib searching	100.00				integral
74	9,12-Octadecadienoic acid (Z,Z)-	C18 H32 O2	31.631		60-33-3	Lib searching	90.08	90.08			integral
75	9,12-Octadecadienoic acid (Z,Z)-	C18 H32 O2	31.757		60-33-3	Lib searching	88.30	88.30			integral
76	9,12,15-Octadecatrienoic acid, (Z,Z,Z)-	C18 H30 O2	31.882		463-40-1	Lib searching	87.98	87.98			integral
77	Octadecanoic acid	C18 H36 O2	32.068		57-11-4	Lib searching	94.30	94.30			integral
78	Hexadecanamide	C16 H33 N O	32.297		629-54-9	Lib searching	92.24	92.24			integral
79	Tricosane	C23 H48	33.835		638-67-5	Lib searching	96.38	96.38			integral
80	4,8,12,16-Tetramethylheptadecan-4-olide	C21 H40 O2	34.626		96168-15-9	Lib searching	95.39	95.39			integral
81	9-Octadecenamide, (Z)-	C18 H35 N O	34.773		301-02-0	Lib searching	92.93	92.93			integral

MassHunter Qual 10.0
(End of Report)
